# Thalassemia Major: how do we improve quality of life?

**DOI:** 10.1186/s40064-016-3568-4

**Published:** 2016-10-28

**Authors:** Nonita Dhirar, Jyoti Khandekar, Damodar Bachani, Deonath Mahto

**Affiliations:** 1 Department of Community Medicine, Lady Hardinge Medical College, New Delhi, India; 2 Department of Pediatrics, Kalawati Saran Children’s Hospital, New Delhi, India

**Keywords:** Beta Thalassemia Major, Quality of life, Hemoglobinopathies

## Abstract

**Background:**

Thalassemia Major is a preventable genetic disorder characterized by abnormal hemoglobin synthesis and lifelong blood transfusions. The children suffering from Thalassemia Major have poor quality of life. This study was conducted to assess the factors influencing quality of life of these children and how it can be improved.

**Methods:**

A descriptive cross sectional study was conducted in 2014 at Thalassemia Day Care Centre of a tertiary level children’s hospital in Delhi, to assess quality of life of children suffering from Thalassemia Major. A total of 241 eligible children (age 2–18 years) were enrolled in the study. Socio demographic and clinical characteristics were collected from interview and existing medical records. The PedsQL 4.0 generic core scale was used for assessing the quality of life of the children.

**Results:**

The mean age of children was 8.69 ± 4.98 years. Two-thirds (63.5%) were boys. The total mean QoL score of the children was 82.0 ± 14.4. The quality of life scores were better for boys as compared to girls. The most affected domain was the emotional domain which showed statistically significant (p = 0.025) difference between boys and girls. The total QoL scores were significantly affected by the current age of the child (p = 0.000) and presence of co-morbidity (p = 0.026). Children not on any form of iron chelation therapy (p = 0.003) and fewer hospital visits (p = 0.044) had better QoL scores.

**Conclusions:**

Factors improving the quality of life were control of iron overload and adverse effects of ICTs, management of co morbidities and fewer hospital visits.

## Background

Thalassemia, the most common single gene disorder in India, is a heterogeneous group of inherited disorders of hemoglobin synthesis. The prevalence of carriers of Thalassemia gene in different regions of India varies from 1 to 17% with a mean prevalence of about 3.3% (Modell and Bulyzhenkov 1988). The severe form of the disease (Thalassemia Major) is characterized by severe anemia requiring repeated blood transfusions, iron overload and disease related complications. The quality of life (QoL) and survival of children suffering from Thalassemia Major are considerably lower as compared to their normal counterparts (Thavorncharoensap et al. 2010). Medical advancement with regular blood transfusion therapy and iron-chelating therapies (ICTs) has dramatically improved the life expectancy of children suffering from Thalassemia Major, (Brittenham et al. 1994; Telfer et al. 2006; Olivieri and Brittenham 1997) but their quality of life remains poor. There are a number of ways through which the quality of life may be improved, a few of which are simple to execute. Very few studies have been conducted on assessment of the modifiable predictors of the same. This study is one such attempt to analyze the factors influencing the quality of life of these children.

## Methods

A descriptive cross sectional study was conducted from November 2013 to March 2015 at Thalassemia Day Care Centre of a tertiary level children’s hospital in Delhi, for assessment of quality of life of children suffering from Thalassemia Major. Data was collected from January 2014 to December 2014 and the compilation, analysis and reporting were done from January 2015 to March 2015. The Thalassemia Day Care center provides free of cost investigations, transfusion facilities, Iron chelation therapy and all supportive medications along with food and leisure activities to the children enrolled at the center. A total of 453 children were enlisted of which 241 were eligible. Children suffering from Thalassemia Major, regularly attending the clinic and whose parents’ consented for the study formed the inclusion criteria. Exclusion criteria were other forms of Thalassemia and refusal to participate in the study. All eligible children (age 2–18 years) diagnosed and registered for treatment and follow-up for Thalassemia Major were enrolled in the study. The children and parents were interviewed in a single session while the children were undergoing transfusion in the centre.

Socio demographic and clinical characteristics were collected from interview and existing medical records. The PedsQL 4.0 generic core scale developed by Varni et al. (1999) was used for assessing the quality of life of the children. This 23-item scale was used to measure the core dimensions of health that is physical, emotional and social, as well as role (school) functioning with developmentally appropriate forms for ages 2–4, 5–7, 8–12 and 13–18 years. Each item is on 5-point rating scales from 0 to 4, labeled “Never/Almost never/Sometimes/Often/Almost always". *Psychosocial Health Summary Score* was computed as the sum of the items over the number of items answered in the Emotional, Social, and School Functioning Scales together. The *Physical Health Summary Score* was the same as the Physical Functioning Scale Score. For the *Total Scale Score,* the mean was calculated by sum of all the items over the number of items answered on all the Scales. Thorough physical examination of the children was done following the administration of questionnaire and the investigations were obtained from the hospital records.

Permission was taken from MAPI Research Institute, Lyon, France prior to using the instrument and Hindi translation of the same was obtained. Written informed consent of the parents/guardians and assent of the children was obtained. The study protocol was approved by Institutional ethical committee of Lady Hardinge Medical College, New Delhi, India.

Data analysis was done using Microsoft excel and Statistical Package for the Social Sciences (SPSS) version 11.5. Data was expressed as mean (SD), median (range) and proportions. The categorical variables were analyzed using Chi square and Fischer’s exact test. The differences between groups were analyzed by unpaired t-test and Mann–Whitney U test. Pearson’s or Spearman’s rank correlation coefficients were used to find out strength of relationships. Multiple regression analysis was done in a stepwise manner to identify independent predictors. All p values were two tailed at a significance level of 0.05.

## Results

All 241 children responded to the questionnaire, giving a response rate of 100%. The mean age of children was 8.69 ± 4.98 years (range: 2–18 years). Two-thirds (63.5%) were boys and almost all (97.9%) were from urban background with majority (71.4%) belonging to Hindu religion. Nearly half (53%) of the subjects belonged to middle socioeconomic status and almost same proportion (53.5%) belonged to nuclear families. Consanguineous marriage was reported by parents of 19.6% of study subjects. About 1/3rd (28.6%) of the subjects reported history of Thalassemia major in the family (Table 1).

**Table 1:** Socio-demographic and clinical characteristics of 241 children suffering from Thalassemia

Socio-demographic and clinical characteristics	n = 241
Mean age (years)	8.69 ± 4.98
Sex	
Male	153 (63.5%)
Female	88 (36.5%)
Religion	
Hindu	172 (71.4%)
Muslim	60 (24.9%)
Others^a^	9 (3.7%)
Socioeconomic status	
Upper	0 (0%)
Middle	122 (53%)
Lower	103 (44.8%)
Not applicable (rural)	5 (2.2%)
Type of family	
Joint	107 (46.5%)
Nuclear	123 (53.5%)
Consanguinity	
Yes	47 (19.6%)
No	194 (80.4%)
Family history of Thalassemia	
Yes	69 (28.6%)
No	172 (71.4%)
Age at onset (months)	
0–6	165 (68.5%)
7–12	40 (16.6%)
13–18	14 (5.8%)
19–24	14 (5.8%)
>24	8 (3.3%)
Presence of co-morbidities/complications	
Yes	17 (7.1%)
No	224 (92.9%)
Iron chelation therapy	
Yes	220 (91/3%)
No	21 (8.7%)

^a^Others included Sikh and Christian

A total of 173 children were of school going age of which 89.5% were currently attending school and 8.6% were school dropouts. A few children (1.7%) were never enrolled in school. The main reasons for dropout were health problems (66.7%) followed by financial constraints (13.3%). Delayed schooling was observed in 42.4% of all the study subjects. Almost 1/3rd (28.3%) of the mothers and 18.3% of the fathers of study subjects were illiterate. Majority (88%) of the mothers were homemakers. Half (49.7%) of the fathers were employed as unskilled and semi-skilled workers while only 5.9% had a professional background.

The mean age at onset of Thalassemia was 8.2 ± 7.1 months (range: 0.5–36 months). The mean age at diagnosis was 9.2 ± 7.4 months (range: 1–36 months). Almost half of the study subjects (45.2%) had hemolytic facies. Delayed puberty was observed in 7.8% of the boys and 5.7% of the girls. Seventeen (7.1%) of the study subjects were suffering from co-morbidities. Majority (88.4%) of the subjects were on only oral iron chelation therapy. Twenty-one (8.7%) of the study subjects were not on any form of iron chelation therapy because of controlled ferritin levels. Side effects were observed in 11.8% of the study subjects on ICT.

The mean health related quality of life (HRQoL) scores are shown in Table 2 which summarizes physical, emotional, social school functioning, psychosocial health and total summary score. The total mean QoL score of the children enrolled at the center was 82.0 ± 14.4. The total quality of life scores were better for boys as compared to girls but this difference was not statistically significant (p = 0.131). The most affected domain was the emotional domain which was better for boys (77.7 ± 15.9) as compared to girls (71.5 ± 19.1) and this difference was statistically significant (p = 0.025).

**Table 2:** Health-related quality of life (HRQoL) of the children with Beta-Thalassemia major

	Total (n = 241)	Boys (n = 153)	Girls (n = 88)	p value*
Physical QoL score	81.3 ± 21.3	82.3 ± 18.7	79.5 ± 25.1	0.121
Psychosocial QoL score	82.5 ± 13.1	83.7 ± 11.8	80.5 ± 14.8	0.079
Emotional domain	75.4 ± 17.4	77.7 ± 15.9	71.5 ± 19.1	0.025
Social domain	92.0 ± 17.4	92.8 ± 15.9	90.6 ± 19.7	0.335
School functioning	77.8 ± 14.5	77.5 ± 15.6	78.2 ± 12.7	0.930
Total QoL score	82.0 ± 14.4	83.1 ± 12.5	80.1 ± 17.2	0.131

Values are expressed as mean (standard deviation; SD), Statistical methods used: Mann–Whitney U test; * p < 0.05 was considered statistically significant

The total QoL scores were significantly affected by the current age of the child (p < 0.001). Presence of co-morbidity significantly altered the quality of life scores across all domains (p = 0.026). Children not on any form of iron chelation therapy had better QoL scores as compared to those on any form (p = 0.003). Fewer visits to the hospital were associated with better quality of life scores (p = 0.044) (Table 3).

**Table 3:** Univariate analysis on covariates associated with HRQoL scores among children with Beta-Thalassemia Major

	Physical QoL Score	p value	Correlation (r, p)	Psychosocial QoL score	p value	Correlation (r, p)	Total QoL score	p value	Correlation (r, p)
Age									
2–4 years (n = 68)	88.9 ± 21.6	0.001	(−0.243, 0.000)	86.1 ± 12.6	0.000	(−0.229, 0.000)	87.4 ± 14.2	0.000	(−0286, 0.000)
5–7 years (n = 43)	83.2 ± 16.4			85.5 ± 10.7			84.5 ± 10.7		
8–12 years (n = 61)	75.7 ± 23.5			77.7 ± 14.3			76.7 ± 15.6		
13–18 years (n = 69)	77.5 ± 19.4			81.5 ± 12.4			79.9 ± 13.6		
Comorbidity									
Yes (n = 17)	67.0 ± 25.9	0.008	(−0.173, 0.007)	76.9 ± 16.6	0.157	(−0.093, 0.150)	72.8 ± 18.5	0.026	(−0.145, 0.024)
No (n = 234)	82.4 ± 20.5			82.9 ± 12.7			82.7 ± 13.9		
Type of Iron chelation therapy									
Only oral (n = 213)	81.2 ± 21.3	0.103		82.0 ± 12.4	0.000		81.7 ± 13.9	0.003	
Only subcutaneous (n = 3)	67.7 ± 17.8			78.3 ± 19.2			74.6 ± 17.2		
Oral +subcutaneous (n = 4)	73.4 ± 14.6			75.4 ± 12.5			74.6 ± 13.0		
No ICT (n = 21)	86.1 ± 21.9			89.7 ± 17.2			88.1 ± 18.6		
Frequency of Hospital visits/month									
1 (n = 196)	83.1 ± 20.4	0.012	(−0.162, 0.009)	82.9 ± 12.9	0.213	(−0.50,0.420)	82.9 ± 14.1	0.044	(−0.127, 0.050)
2 (n = 43)	74.6 ± 23.2			81.3 ± 13.5			78.7 ± 15.4		
3 (n = 2)	51.6 ± 6.6			69.2 ± 1.2			63.0 ± 3.07		

Values are expressed as mean (standard deviation; SD), Statistical methods used: Mann–Whitney U test for difference between groups, Correlation coefficient measured for correlation; * p < 0.05 was considered statistically significant

A strong negative correlation of the physical domain scores was observed with current age of the child. Younger patients had higher total summary scores as compared to older patients (p < 0.001). Based on clinical characteristics, age at onset (p = 0.017), frequency of transfusions per month (p = 0.009), treatment duration (p < 0.001), number of concomitant medicines (p = 0.011) and co morbidities (p = 0.007) were significantly associated with self reported Physical summary scores (Table 3).

A multiple linear regression was run to predict physical quality of life scores from current age of the child, age at onset, number of concomitant medications and co-morbidities, frequency of transfusions per month and duration of treatment. These variables together statistically significantly predicted physical quality of life (R = 0.325, R^2^ = 0.106, p = 0.000). 10.6% of all variation in the physical quality of life scores was due to these independent variables (Table 4).

**Table 4:** Multiple linear regression analysis on predictors of the HRQoL scores on subscales of Peds QoL reports

	R	R^2^	F (DF, P)	β coefficient	p value*
Physical QoL	0.325	0.106	4.617 (6, 234)	105.468	0.000
Age in completed years				0.459	0.647
Age of onset in months				−1.913	0.057
Number of comorbidities				−1.665	0.097
Duration of treatment in years				−0.687	0.493
Frequency of transfusion per month				−1.959	0.051
Number of Concomitant medications				−1.510	0.132
Psychosocial QoL	0.211	0.044	3.663 (3, 237)	88.327	0.013
Age in completed years				−2.309	0.207
Duration of treatment				1.432	0.441
Total number of visits per year				−0.101	0.793
Total QoL	0.310	0.096	3.536 (7, 233)	97.264	0.001
Age in completed years				−0.012	0.994
Age of onset in months				−0.280	0.109
Frequency of transfusion per month				−2.529	0.305
Duration of treatment in years				−0.408	0.806
Number of Concomitant medications				−2.193	0.213
Total number of visits per year				−0.093	0.741
Number of comorbidities				−4.902	0.094

Statistical method used: multiple linear regression; * p < 0.05 was considered statistically significant

Psychosocial domain scores strongly correlated with age in completed years (p = 0.000), duration of treatment (p = 0.001) and number of visits per year (p = 0.011). Multiple linear regression predicted that about 4.4% of the variation in the psychosocial quality of life score was due to these three variables. The variables statistically significantly predicated the scores. (R = 0.211, R^2^ = 0.044, p < 0.001) as shown in Table 4.

The mean scores of total quality of life scores were negatively predicted by current age of the child (p < 0.001), age at onset (p = 0.025), frequency of transfusions (p = 0.050), duration of treatment (p < 0.001), total number of visits per year (p = 0.006), number of concomitant medicines (p = 0.013) and number of co-morbidities (p = 0.024). The Multiple linear regression run between these variables and total quality of life scores showed that these variables statistically significantly predicted the total quality of life scores, (R = 0.310, R^2^ = 0.096, p = 0.001). 9.6% of the variation in the total quality of life scores was due to these variables (Table 4).

## Discussion

The QoL in the present study was measured using PedsQL 4.0 generic core scales. A normal child should have a score of or closer to 100 for all domains in this questionnaire. The quality of life scores of the 241 children in the present study were low as compared to the normal children (total summary score: 82.0 ± 14.4, psychosocial summary score: 82.5 ± 13.1 and physical health score: 81.3 ± 21.3).

Scores across all domains were higher in present study as compared to other studies (Ayoub et al. 2013; Caocci et al. 2012; Gharaibeh and Gharaibeh 2012; Wahyuni et al. 2011; Surapolchai et al. 2010; Clarke et al. 2010; Ismail et al. 2006). This may be attributed to the better access to health care facilities and better control of iron overload related complications at this tertiary care center. The parents and children are informed about the next transfusion beforehand which is calculated as per their rate of fall in hemoglobin level. The children are thoroughly assessed for iron overload and co-morbidities at every visit by a resident doctor and any investigation and intervention required is done as soon as possible, free of cost.

The total summary scores were higher among boys in our study as compared to girls though the difference was not statistically significant. The lower scores in girls may be due to poor attention given to girl child in our society. On the contrary, some studies (Caocci et al. 2012; Saha et al. 2015) have reported better scores for girls and other studies have shown no difference between boys and girls (Thavorncharoensap et al. 2010; Gharaibeh and Gharaibeh 2012; Torcharus and Pankaew 2011).

Age was an important predictor for all the three summary scores of the quality of life questionnaire. One explanation for the higher scores in younger ages is the recent onset of disease resulting in less iron overload and hence lesser complications. A rise in the quality of life scores in all domains was observed in the age group of 13–18 years after a dip in the age group of 8–12 years. The observation is probably due to the process of adaptation of the teenage group to the lifestyle associated with Thalassemia.

Children who had any form of co morbidity had poorer total quality of life scores as compared to those who did not (p < 0.001). Absence of co morbidities improves the overall quality of life. Co-morbidities affect both the physical and psychological quality of life severely. The number of co morbidities was a strong predictor of the poor physical and total quality of life score (Table 4).

Introduction of iron chelation therapy (ICT) has been a boon in the management of Thalassemia but it has its own pros and cons. Only 8.7% of the children in the present study were not on any form of iron chelation therapy (ICT). The rest were on either oral or subcutaneous or both forms of ICT. The total quality of life scores were better for children who were not on any form of ICT (p = 0.003), since the children were not exposed to ICT associated side effects like nausea, pain in joints, and pain at the site of injection (in case of subcutaneous therapy) which hamper the normal day to day activities of the children. Children on oral iron chelation therapy had higher scores compared to those on combined oral and subcutaneous therapy. The painful subcutaneous injections greatly affect the psychosocial health. Torcharus et al. (2011) reported no difference in QOL based on type of iron chelation therapy while Surapolchai et al. (2010) and Thavorncharoensap et al. (2010) reported significant difference in quality of life scores due to iron chelation therapy.

Frequency of blood transfusion and the total number of visits per year were predictors of physical and total quality of life scores and negatively affected QoL scores in the present study. Children with 1 transfusion per month had better total QoL scores as compared to children visiting 3 times a month for transfusion. More frequent visits to the hospital have negative impact on children’s lives in terms of physical burden, psychological burden and school attendance thus affecting the quality of life. Children have a constant stress of travelling all the way from long distances and being subjected to painful investigations and transfusion procedures. This finding was comparable to that reported by Surapolchai et al. (2010) while Caocci et al. (2012) reported no relation between visits and HRQoL scores.

The independent variables for predicting the total quality of life score viz current age, age at onset, frequency of transfusions, duration of treatment, total number of visits per year, number of concomitant medicines and number of co-morbidities predicted only 9.6% of the variation in the total quality of life scores. Some of these factors like age of the child, age of onset are non-modifiable, while rest can be improved by regular treatment and better clinical management as indicated by the study. On the contrary Saha et al. (2015) showed variation in total QoL score due to variables like duration since splenectomy, last pre transfusion Hb level and family history of thalassemia, which were not significant predictors in the present study.

Apart from clinical management of the disease, another major factor affecting the overall quality of life of the children is the availability of health care services free of cost. Every service right from the blood transfusion kit to the cost of travel drains a lot of money from the family savings. A big contribution of the center is the provision of free blood, drugs, food, passes for travel and investigations which has been a major contributory factor for the overall improvement of quality of life.

## Conclusion

Thalassemia is a disease which impairs the quality of life of children significantly. Major factors affecting the quality of life are related to better control of iron overload and adverse effects of ICTs, prevention of co morbidities, fewer hospital visits and good clinical management. Early diagnosis, regular monitoring, education and financial support are the major areas of intervention to improve the overall quality of life of these children.

## Abbreviations

HRQoL: Health related quality of life
QoL: Quality of life
ICT: Iron chelation therapy
SPSS: Statistical Package for the Social Sciences
SD: Standard deviation
